# The relationship between physical and mental health multimorbidity and children’s health-related quality of life

**DOI:** 10.1007/s11136-022-03095-1

**Published:** 2022-01-29

**Authors:** Rachel O’Loughlin, Harriet Hiscock, Tianxin Pan, Nancy Devlin, Kim Dalziel

**Affiliations:** 1 Health Economics Unit, Centre for Health Policy, Melbourne School of Population and Global Health, University of Melbourne, Melbourne, VIC 3010 Australia; 2 Department of Paediatrics, Melbourne Medical School, University of Melbourne, Melbourne, VIC 3010 Australia; 3 Health Services Research Unit, The Royal Children’s Hospital, Parkville, VIC 3052 Australia; 4 Health Services, Murdoch Children’s Research Institute, Parkville, VIC 3052 Australia

**Keywords:** Quality of life, Pediatrics, Chronic disease, Mental health, Multimorbidity

## Abstract

**Purpose:**

To examine the relationships between physical health problems, and borderline or clinical levels of mental health symptoms and children’s health-related quality of life (HRQoL).

**Methods:**

Data were from the *Longitudinal Study of Australian Children* (2004–2018). Parents reported on their child’s HRQoL (PedsQL), physical health problems and mental health symptoms (Strengths and Difficulties Questionnaire, SDQ). A pooled cross-sectional analysis using linear regressions examined the relationships between physical health and clinical/borderline mental health symptoms, individually and when multi-morbid, and children’s HRQoL, and whether these relationships vary by a range of child, family and social factors.

**Results:**

The sample comprised 47,567 observations of children aged 4–17 years. Borderline and clinical levels of mental health symptoms were associated with significantly lower HRQoL, equal to more than two-times (10.5 points) and more than three-times (16.8 points) the clinically meaningful difference, respectively. This was a larger difference than that associated with physical health problems (4.4 points). We found a significant interaction effect between physical health problems and clinical mental health symptoms which was associated with even poorer HRQoL after accounting for the individual relationships of both problems. Mental health problems were associated with poorer HRQoL for older versus younger children; and the interaction effect was significant for boys but not girls.

**Conclusion:**

Findings highlight the importance of identifying and addressing mental health symptoms in children of all ages, even if these problems do not meet formal clinical criteria. Particular attention should be paid to the mental health and HRQoL of children with physical–mental multimorbidity, who are at risk of disproportionately poorer HRQoL.

**Supplementary Information:**

The online version contains supplementary material available at 10.1007/s11136-022-03095-1.

## Plain language summary

Ongoing physical and mental health problems are common in children and adolescents and, often, children can experience both problems together. Mental and physical health problems can have wide impacts for the child, including their health-related quality of life (HRQoL), which is a measure of the way the child’s health impacts their emotional, social and physical functioning during their day-to-day life. Our study shows that children with high levels of mental health symptoms have much poorer HRQoL than their peers, and we provide new evidence that even milder mental health symptoms are associated with poorer HRQoL than in children with physical health problems. When children have both physical and mental health problems, they are at even greater risk of poorer HRQoL than would be expected. Based on our findings, we recommend that clinicians should monitor and address mental health symptoms in children as young as 4–7 years old, even if these symptoms are milder, and particular attention should be given to children with physical and mental health problems, who are at greater risk of poor HRQoL.

## Introduction

It has been suggested that quality of life is the “*universal outcome towards which all our efforts regarding children ultimately should be directed* [1].” Indeed, there has been a shift in focus from *quantity* of life years towards the *quality* of those years. International prevalence rates vary, though it has been estimated that approximately half of American children experience a chronic condition during childhood [2]. In Australia, 60–72% of children aged 2–15 years are experiencing an ongoing health condition; commonly obesity, asthma, and eczema [3] or mental health conditions such as Attention-Deficit/Hyperactivity Disorder (ADHD), anxiety disorders and depression [4]. These physical and mental health problems frequently co-occur [5, 6], defined as physical–mental multimorbidity [7], however, our understanding of the quality of life of children with physical–mental multimorbidity remains limited in comparison to our understanding in adults [6].

Health-related quality of life (HRQoL) is a multi-dimensional measure of a person’s subjective thoughts and feelings regarding the impact of their health status across various aspects of their life, typically including physical, social and psychological domains [8]. It is well-established that children with a range of physical health problems have lower HRQoL than their healthy peers [9]. Fewer studies have examined HRQoL in children with mental health conditions, but results show poorer HRQoL both compared to their healthy peers [10, 11], and to those with physical health problems [9, 12–14].

‘Subthreshold’ (or borderline) levels of mental health symptoms are those that do not meet formal clinical criteria. Borderline symptoms are common in children and adolescents [15], and show a similar risk profile to those with clinical symptoms in terms of healthcare service use; functional impairment; psychiatric comorbidity; risky drug use and suicidality [16, 17]. One study found HRQoL in these children was intermediate between children with clinical symptoms and those with lower levels of mental health symptoms [17]. This is in line with dimensional analyses that have found greater mental health symptoms are related to increasingly poorer HRQoL [18–20]. To our knowledge no studies have compared the relative relationships between children’s HRQoL and borderline and clinical mental health symptoms, and physical health problems, which is crucial if we are to understand which children are at greater risk of poor HRQoL.

A number of studies have examined the relationships between physical–mental multimorbidity and children’s HRQoL, with mixed results. For example, some have found multimorbidity is associated with poorer overall HRQoL than a single physical or mental health condition [12, 13, 21]; though others have noted poorer HRQoL only when children had three or more conditions [22], or increasing numbers [23] of mental health conditions. In contrast, others have found no significant association between HRQoL and comorbid physical health problems after accounting for the relationship with existing mental health problems [20]. Most existing studies focus on multimorbidity within a single physical health disorder (most commonly epilepsy, asthma or allergies); and most estimates were based on small, observational studies from North America and Europe [6].

Only one study has formally examined the interaction effect of physical–mental multimorbidity on children’s HRQoL. Sawyer et al. [12] reported a significant interaction effect—i.e. HRQoL was poorer than the additive effect of mental and physical health conditions—in children aged 6–17 years in Australia. However, this study was conducted almost 20 years ago and its finding was based on a small multimorbid sample (*n* = 80) and a limited set of mental health conditions (70% ADHD, 19% major depressive disorder and 11% conduct disorder). In addition, the study excluded children with more than one mental health condition which may lead to an underestimation[24], and did not examine the relationships between children’s HRQoL and wider parental and social factors such as parent mental illness, parenting stress and socioeconomic status that have previously been found [20, 25].

The existing evidence leaves a notable gap in our understanding of the HRQoL of children with mental health disorders, particularly those with borderline symptoms, and the interaction of mental and physical health problems on children’s HRQoL. We therefore aimed to examine (1) the relative relationships between HRQoL and clinical and borderline levels of mental health symptoms, and physical health symptoms; (2) the interaction effect of physical–mental multimorbidity on HRQoL and (3) whether these relationships vary by a range of child, parent, family and social factors.

## Methods

### Data & ethics approval

Data were pooled from eight waves of the *Longitudinal Study of Australian Children* (LSAC); a nationally representative sample of Australian children followed every 2 years between 2004 and 2018 [26]. The LSAC study was approved by the Australian Institute of Family Studies Ethics Committee and families provided written consent to participate. The current study was approved by the data custodians and no further ethics approval was required.

### Participants

Participants were children aged ≥ 4 years and < 18 years at the time of data collection, and their primary caregiver. This age range was chosen to match the recommended age range for the measures described below, and data availability within the LSAC study.

### Measures

#### Primary outcome measure

*Children’s health-related quality of life (HRQoL)* was measured using the Pediatric Quality of Life Inventory (PedsQL), a 23-item generic measure of HRQoL comprising four domains: physical; emotional; social and school functioning [27]. The PedsQL is feasible, valid and reliable in general population research [27]. Parents rated their child’s functioning over the last month on a scale from 1 ‘never a problem’ to 5 ‘almost always a problem’. Items were reverse scored and linearly transformed to a 0–100 scale. A total score was calculated as the sum of each item divided by the number of items answered at each time point, provided less than half of the items were missing [27]. Higher scores represent better HRQoL, and a 4.5 point change in the PedsQL total score is considered a clinically meaningful difference using the parent proxy-report form [27].

#### Independent variables

*Physical health problems* Parents reported whether their child had any ‘ongoing condition(s)’ (defined in LSAC as conditions that exist for some period of time—weeks, months or years—or re-occur regularly; they do not have to be diagnosed by a doctor). See Table 1 for full list of included conditions. The child’s height and weight were recorded, and BMI calculated. Additionally, parents responded yes/no to a special healthcare needs (SHCN) screening question: “*Child has a condition which has lasted or is expected to last for at least 12 months which causes them to use medicine prescribed by a doctor (other than vitamins) or more medical care, mental health or educational services*”. Children were included in the ‘physical health problem’ group if their parent reported at least one ongoing physical health problem; OR the child’s weight status was overweight or obese based on the BMI threshold relevant for their age (see Cole et al. [28]); AND the parent answered ‘yes’ to the SHCN screening question. This narrow definition was used to ensure the child’s problems were serious enough to warrant ongoing medical care and could reasonably be expected to be related to differences in HRQoL.

**Table 1:** Sample characteristics for total sample and separate problem groups

Sample characteristics	Total sample	Breakdown by problem area			
		Not classified as PH or MH	Physical health + SHCN (PH)	Mental health (MH)	Multimorbid PH + MH
Total number of observations	47,567	34,599	7251	8296	2579
*Mental health breakdown*					
Mental health problems defined by SDQ					
Borderline, total score 13–16*, n* (%)	4732 (10.0)	–	1092 (15.1)	4732 (57.0)	1092 (42.3)
Clinical, total score 17 + , *n* (%)	3564 (7.5)	–	1487 (20.5)	3564 (43.0)	1487 (57.7)
Internalising problems, clinical, *n* (%)	3152 (6.6)	219 (0.6)	1316 (18.2)***	2852 (34.4)***	1235 (47.9)***
Externalising problems, clinical, *n* (%)	4324 (9.1)	387 (1.1)	1433 (19.8)***	3874 (46.7)***	1370 (53.1)***
Mental health problems defined by parent-reported condition					
Parent-reported anxiety and/or depression, *n* (%)	2074 (6.4)	533 (2.3)	1068 (19.9)***	1233 (22.4)***	760 (39.3)***
Parent-reported ADHD, *n* (%)	1161 (2.4)	126 (0.4)	815 (11.2)***	891 (10.7)***	671 (26.0)***
*Physical health breakdown*					
Special healthcare needs, *n* (%)	7987 (16.8)	469 (1.4)	*All*	2846 (34.3)	*All*
Any physical health, *n* (%)	33,677 (70.8)	22,265 (64.4)	*All*	6740 (81.2)	*All*
Asthma	13,542 (28.5)	7402 (21.4)	4609 (63.6)***	2961 (35.7)***	1430 (55.5)***
Overweight, obesity status	10,938 (23.0)	7440 (21.5)	2063 (28.5)***	2215 (26.7)***	780 (30.2)***
Had cavities or dental decay (last 2 years)	8933 (18.8)	6132 (17.7)	1641 (22.6)***	1836 (22.1)***	676 (26.2)***
Eczema	5283 (11.1)	3054 (8.8)	1684 (23.2)***	1017 (12.3)***	472 (18.3)***
Hearing or vision	5102 (10.7)	2996 (8.7)	1405 (19.4)***	1299 (15.7)***	598 (23.2)***
Recurrent pain (inc headaches)	4197 (8.8)	2185 (6.3)	1300 (17.9)***	1328 (16.0)***	616 (23.9)***
Moderate to large sleep problems	4033 (8.5)	1755 (5.1)	1365 (18.8)***	1795 (21.6)***	882 (34.2)***
Hay fever	2438 (5.1)	1437 (4.2)	817 (11.3)***	420 (5.1)***	236 (9.2)***
Bone, joint or muscle problems	2362 (5.0)	1367 (4.0)	683 (9.4)***	572 (6.9)***	260 (10.1)***
Recurrent infections (ear or tonsillitis)	2333 (4.9)	1345 (3.9)	601 (8.3)***	651 (7.9)***	264 (10.2)***
Constipation, diarrhoea, colitis or irritable bowel	1463 (3.1)	667 (1.9)	574 (7.9)***	524 (6.3)***	302 (11.7)***
Food or digestive allergies	1218 (2.6)	622 (1.8)	451 (6.2)***	303 (3.7)***	158 (6.1)***
Soiling, day wetting, bed wetting	514 (1.1)	168 (0.5)	272 (3.8)***	259 (3.1)***	185 (7.2)***
Acne	442 (0.9)	243 (0.7)	175 (2.4)***	68 (0.8) *n.s*	44 (1.7)***
Epilepsy or seizure	183 (0.4)	20 (0.1)	152 (2.1)***	97 (1.2)***	86 (3.3)***
Diabetes	110 (0.2)	2 (0.01)	108 (1.5)***	18 (0.2)***	18 (0.7)***
Chronic fatigue	73 (0.2)	12 (0.03)	51 (0.7)***	37 (0.5)***	27 (1.1)***
Congenital heart condition	37 (0.1)	5 (0.01)	26 (0.4)***	17 (0.2)***	11 (0.4)***
*Child characteristics*					
Child age in years, *mean (SD)*	10.1 (3.7)	10.1 (3.7)	10.7 (3.8)***	9.8 (3.8)***	10.6 (3.4)***
Male, *n* (%)	24,363 (51.2)	16,893 (48.8)	4140 (57.1)***	4953 (59.7)***	1623 (62.9)***
Number of chronic conditions, *mean (SD)*	1.4 (1.4)	1.1 (1.1)	2.7 (1.6)***	2.0 (1.7)***	3.0 (1.9)***
SDQ total score, *mean (SD)*	7.8 (5.4)	5.7 (3.2)	10.7 (6.8)***	16.9 (3.8)***	18.4 (4.5)***
PedsQL total score, *mean (SD)*	80.3 (13.1)	83.4 (10.9)	73.2 (15.7)***	67.7 (14.3)***	62.1 (14.8)***
CHU9D utility score, *mean (SD)*	0.80 (0.20)	0.82 (0.18)	0.75 (0.22) ***	0.73 (0.24) ***	0.70 (0.25) ***
*Family and neighbourhood characteristics*					
Parent sex, female, *n* (%)	46,574 (97.9)	33,892 (98.0)	7091 (97.8) *n.s*	8117 (97.8) *n.s*	2526 (97.9) *n.s*
Main language at home, LOTE, *n* (%)	4249 (8.9)	3106 (9.0)	445 (6.1)***	840 (10.1)**	142 (5.5)***
Parental distress, ‘*probable serious mental illness’*, *n* (%)	1334 (2.8)	579 (1.7)	356 (4.9)***	643 (7.8)***	244 (9.5)***
Parent 1 completed higher education, *n* (%)	36,958 (77.8)	27,119 (78.5)	5750 (79.4) *n.s*	6051 (73.0)***	1962 (76.2)**
Single parent household, yes, *n* (%)	6942 (14.6)	4483 (13.0)	1234 (17.0)***	1864 (22.5)***	639 (24.8)***
Number of siblings, *mean (SD)*	1.5 (1.0)	1.6 (1.0)	1.4 (1.0)***	1.5 (1.1)**	1.4 (1.0)***
Maladaptive parenting, yes, *n* (%)	2818 (6.0)	1320 (3.8)	560 (7.8)***	1338 (16.4)***	400 (15.8)***
Annual household income $AUD, *mean (SD)*	115,807.5 (85,702.8)	119,723.5 (88,017.4)	114,083.5 (82,447.8)***	95,819.3 (73,560.2)***	100,586.2 (79,831.5)***
SEIFA, IRSAD, *mean (SD)*	1012.8 (74.7)	1016.5 (74.0)	1009.9 (74.7)***	994.9 (75.5)***	997.2 (75.4)***
Remoteness—resides in ‘Major City’, *n* (%)	30,448 (64.1)	22,194 (64.2)	4706 (65.0) *n.s*	5156 (62.2)**	1608 (62.4) *n.s*

*CHU9D* Child Health Utility instrument, *IRSAD* Index of relative socioeconomic advantage and disadvantage, *LOTE* language other than English, *MH* mental health, *PedsQL* Pediatric Quality of Life Inventory, *PH* physical health (defined as physical health problem plus special healthcare needs), *SDQ* Strengths and Difficulties Questionnaire, *SEIFA* Socioeconomic Indexes for Area, *SHCN* special healthcare needs

*n.s.,* *, **, ***not significant, or significantly different to “no PH or MH” group at *p* < 0.05 *p* < 0.01 and *p* < 0.001, respectively

*Mental health problems* were measured using the Strengths and Difficulties Questionnaire (SDQ), a widely used screening instrument for assessing behavioural and emotional problems in children [29], with five subscales: emotional, peer, behavioural and hyperactivity problems and prosocial behaviours. Parents rated their child’s behaviour over the past 6 months on a scale from 0 ‘Not True’ to 2 ‘Certainly True’. The measure has moderate to strong internal reliability and adequate validity in community samples of Australian children [30, 31]. Higher scores reflect greater problems. Based on Australian norms [31], a total score of 13–16 (out of 40) indicates ‘borderline’ symptoms, and scores ≥ 17 indicate clinically elevated symptoms.

Children with physical–mental multimorbidity were defined as those with at least one physical health problem and SHCN, and either borderline or clinical mental health symptoms.

#### Covariates

*Child age *(*in years*) was recorded at each wave and was included as a categorical variable ‘4–7 years’; ‘8–12 years’ and ‘13–17 years’ to align with the use of the various developmentally appropriate forms of the PedsQL in each of these age bands. *Child sex* was recorded at Wave 1, and this value was continued to other waves.

We additionally adjusted for a range of child, parent, family and social factors that have previously been linked to HRQoL [20, 25], or are hypothesised to be linked in line with Bronfenbrenner’s model of the sociocultural influences on children’s lives [32]. This included *parent mental illness*, which was measured using the Kessler 6 [33], a six item screening scale that asked the primary caregiver to rate their own feelings of sadness and worry over the past 4 weeks on a scale from 1 ‘all the time’ to 5 ‘none of the time’. Scores were reverse coded such that higher scores reflect greater problems and were dichotomised in line with the recommended cut-points: a total score of 6–18 suggests ‘No probable serious mental illness’ and 19–30 suggests ‘Probable serious mental illness’. This variable was included for the primary carer (largely the child’s mother), who also provided the proxy-report of the child’s mental health symptoms (SDQ); physical health problems and HRQoL (PedsQL).

In line with previous research [34], we used a composite measure to indicate m*aladaptive parenting,* combining low warmth and high hostility in parenting behaviours. *Parental warmth* was measured as the mean of six items, e.g. “How often do you express affection by hugging, kissing and holding this child”. *Parental hostility* was measured as the mean of six items, e.g. “How often do you tell this child that he/she is bad or not as good as others”. Both were rated on a 5-point scale from 1 ‘never/almost never’ to 5 ‘all the time’, such that higher scores represent higher levels of parenting warmth and hostility. Mean scores were divided into quintiles, and *maladaptive parenting* was indicated when parents concurrently scored in the highest quintile for *hostility* and the lowest quintile for *warmth*.

Other covariates included *child ethnicity*; *parent education*; *single parent household*; *number of siblings*; *household income*; *socioeconomic status* and *rurality of home postcode*. See Supplementary Table 1 for full details of all covariates.

### Statistical analyses

LSAC cross-sectional population weights were applied to each wave to account for the unequal probability of participant selection and sample attrition so that the individually weighted waves are population representative [35]. This approach of utilising the cross-sectional characteristics of LSAC survey allows us to investigate a population-representative sample with individual respondents that each have different representation across each wave based on the weights applied. Complete case analysis was undertaken. Due to the large sample size, a conservative significance level of *p* < 0.0001 was used. All data were analysed using StataSE 16 (Statacorp, Texas, US).

Sample characteristics were explored using descriptive statistics for the total sample, and for children who had (1) physical health problems and SHCN; (2) borderline or clinical mental health symptoms and (3) physical–mental multimorbidity. Group differences were explored using t-tests for continuous variables and *χ*^2^ for categorical variables comparing each of these problem groups to children not classified into these problem groups.

For regression analyses, we explored the distribution of the error terms using a Modified Park Test, and the results suggested an Ordinary Least Squares (OLS) linear regression model performed well. Firstly, we conducted a regression of the dependent variable (HRQoL) on independent variables (presence/not of borderline or mental health symptoms; or any physical health problem) including the interaction terms between borderline/clinical mental health symptoms and physical health problems. Secondly, we repeated the analysis adjusting for all covariates described above, examining the individual associations between HRQoL and each variable holding all others constant. Thirdly, we repeated the adjusted regression analysis within subgroups of child age and sex.

We conducted a range of sensitivity analyses to examine the robustness of the relationships under alternate definitions of physical health problems, mental health symptoms and HRQoL, in a series of adjusted regressions. See Supplementary Table 2 for full details of the definitions tested and measures used. Of note, as per previous research and recommendations regarding HRQoL measurement in mental health populations [10, 36, 37], our sensitivity analyses tested whether any observed relationship between mental health symptoms and HRQoL was due to item overlap between the two measures. Additionally, due to the known differences and challenges associated with using parent proxy-report versus child self-report of HRQoL [8, 10, 38] we conducted a sensitivity analysis using child self-reported HRQoL measured via the Child Health Utility instrument (CHU9D) [39]. This was included as a sensitivity analysis rather than the main analysis as the child’s self-report was only available in Waves 6–8.

## Results

### Sample characteristics

The total number of observations that met inclusion criteria for age ≥ 4 years and < 18 years was 51,501 across the eight waves. Observations from each wave were dropped if they had insufficient PedsQL (*n* = 3645) or SDQ data (*n* = 1943); or if they were missing SHCN information (*n* = 610). Total excluded cases were *n* = 3934, leaving 47,567 observations (*n* = 9270 unique children, 92.4% of original sample) included in the final analyses. We present the participant flowchart in Supplementary Fig. 1. A larger proportion of excluded observations were for children who were aged 4–7 years (65.8% vs 32.6%); spoke a language other than English (20.0% vs 8.9%); and met the clinical cut-off on the SDQ (11.1% vs 7.5%) and fewer excluded observations had a physical health problem (64.0% vs 70.8%). Missingness of covariates was < 1.0%, except for income (6.7%).

Table 1 shows the sample characteristics overall, and for each problem group. Overall, the mean age was 10.1 years (SD 3.7) and 51.2% were males. The majority of the sample (72.7%) were observations not classified into one of the problem groups, whereas 15.2% had at least one physical health problem and SHCN; 17.4% had borderline or clinical mental health symptoms and 5.4% of observations were children with physical–mental multimorbidity. Significant differences were observed between each problem group and those not otherwise classified for nearly all sample characteristics except for parent sex (97.9% were mothers), and proportion residing in a major city (64.1%).

### Unadjusted and adjusted OLS regression results

**Fig. 1:**
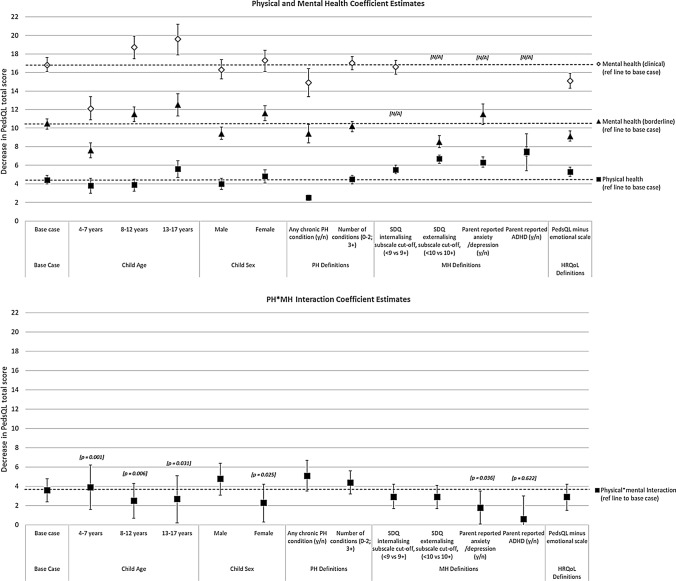
Comparative results of adjusted base case, subgroup and sensitivity analyses. *HRQoL* health-related quality of life, *MH* mental health, *PedsQL* Pediatric Quality of Life Inventory, *PH* physical health, *SDQ* Strengths and Difficulties Questionnaire. All *p* values are *p* < 0.0001 unless otherwise specified. Coefficients are expressed as positive values, representing the *absolute decrease* in PedsQL scores. Results provided for coefficient estimates for PH, MH and MH*PH interaction terms. For base case and subgroup analyses (age and sex), PH, MH and HRQoL are defined as per the main analyses. In the alternate definition analyses, only PH or MH or HRQoL definitions are changed (as listed on the *x*-axis and described in Supplementary Table 2), holding other definitions constant. Where ‘N/A’ is listed, this definition of MH resulted in only one MH coefficient term (i.e. yes vs no; clinical cut-off vs not); the results of these analyses are therefore only listed in the figure once alongside either the borderline or clinical estimates

Table 2 shows results for unadjusted and adjusted regression models. In both analyses, physical and mental health problems were each associated with significantly lower ratings of HRQoL, however, only mental health problems were associated with a clinically meaningful difference in HRQoL. In adjusted analyses, though slightly attenuated, the associations between borderline and clinical mental health symptoms and HRQoL remained equal to more than two-times (10.5 points) and more than three-times (16.8 points) the clinically meaningful difference, respectively. A statistically significant interaction effect was observed for children with physical–mental multimorbidity, where children with clinical (but not borderline) levels of mental health symptoms had lower ratings of HRQoL than the additive effect of mental and physical health problems. Of note, when the primary carer screened positive for their own ‘*probable serious mental illness’*, children showed an additional significant and clinically meaningful difference in HRQoL (4.5 points lower), after accounting for all other factors Fig. 1 shows results of adjusted regression analyses alongside results of each subgroup and sensitivity analysis (see Supplementary Table 3 for data tables for Fig. 1).

**Table 2:**
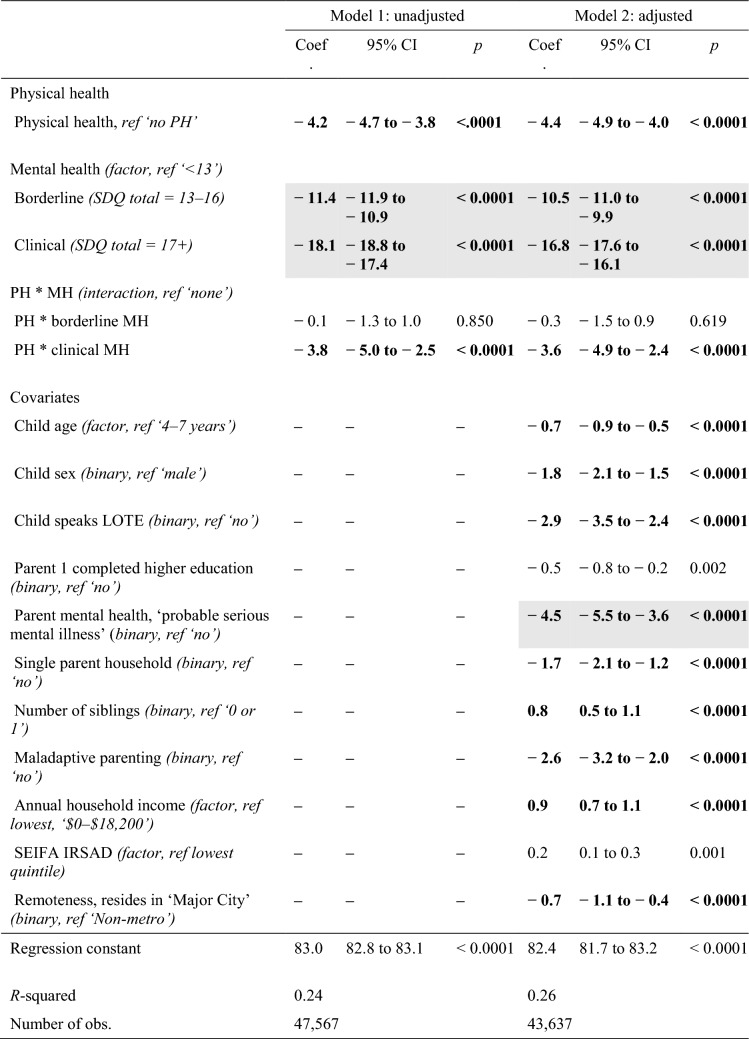
Unadjusted and adjusted relationships between health-related quality of life, and mental health and physical health symptoms

*IRSAD* Index of relative socioeconomic advantage and disadvantage, *LOTE* language other than English, *MH* mental health, *PH* physical health, *SDQ* Strengths and Difficulties Questionnaire, *SEIFA* Socioeconomic Indexes for Area

NB: Bolding denotes statistically significant effects at *p* < .0001; Shading denotes statistically significant and clinically meaningful differences

### Subgroup analyses: child age & sex

The same pattern of results was observed for subgroup analyses by child age and sex (Fig. 1, and Supplementary Table 4), with differences in HRQoL associated with borderline and clinical symptoms ranging from 7.6–12.5 points lower and 12.1–19.6 points lower, respectively. Lower HRQoL was generally observed for older (8–12 and 13–17 years) compared to younger children (4–7 years). Physical health problems were only associated with a significant and clinically meaningful difference in HRQoL in the oldest age band (13–17 years; 5.6 points lower) and for girls (4.8 points lower). The interaction of physical health and clinical mental health problems was significant for boys, but not girls (see Supplementary Fig. 2), and was not significant in each age band individually.

### Sensitivity analyses

Relationships between physical and mental health problems and HRQoL were similar to the main analysis when examining relationships within individual domains of the PedsQL (Supplementary Table 5). As would be expected, there was a significant and clinically meaningful relationship between mental health problems (both borderline and clinical levels) and the emotional functioning domain. Importantly, however, this relationship was additionally observed in the domains of social functioning and physical health. Clinical levels of mental health symptoms were additionally associated with a clinically meaningful difference in school functioning. Reflecting the main analysis, physical health problems were associated with a significant, but not clinically meaningful, difference in HRQoL across all domains. The interaction of physical health and clinical mental health problems was significant within the domains of emotional functioning and social functioning.

When using child-reported HRQoL (CHU9D) as the outcome, we found a similar pattern of results as with parent-report regarding the relative relationships between clinical and borderline mental health problems and physical health problems. However, in contrast to parent-report, there was no significant interaction effect of physical and mental health problems. Additionally, the model explained less variance in child self-reported HRQoL scores (7%) compared to parent-reported HRQoL (26%).

Results of further sensitivity analyses regarding alternative definitions of physical health, mental health and HRQoL are presented in Fig. 1 (see also Supplementary Tables 3 and 6). Findings were similar to the main analysis, with the exception that the interaction of physical health and clinical mental health problems was no longer significant when mental health was defined as ‘*parent report of anxiety and/or depression*’ or *‘parent report of ADHD’*.

## Discussion

### Main findings

Children with borderline or clinical levels of mental health symptoms each had lower ratings of HRQoL than those with physical health problems, whilst children with physical–mental multimorbidity showed even lower ratings over-and-above the additive effect of both problems. Mental health problems were associated with poorer HRQoL for older compared to younger children, though were still associated with a significant and clinically meaningful difference in children aged 4–7 years. In subgroup analyses, the interaction effect was significant for boys only, meaning that boys with physical–mental multimorbidity are at particular risk of disproportionately poorer HRQoL than boys with either mental or physical health problems alone.

In line with previous research we found children with clinical levels of mental health symptoms have poorer HRQoL than children with physical health problems [9, 12–14], and that greater mental health problems are associated with poorer HRQoL [18–20]. Our study provides novel evidence that quantifies the relative relationships between HRQoL and borderline mental health symptoms versus physical health problems, and showed that borderline mental health symptoms are also associated with a clinically important difference in children’s HRQoL that is larger than that of physical health problems. This is important in determining groups of children in greater need for support.

Our finding that mental health problems were associated with lower ratings of HRQoL in older compared to younger children has been noted previously for children with depression (as rated by their parents) [13], though we extended this knowledge to include borderline symptoms. This finding may be due to greater disease severity [14] or greater psychiatric comorbidities [13] in adolescents. Yet others have noted no difference by child age [9, 40]. Longitudinal studies examining the onset and progression of mental health problems are warranted to disentangle these mixed findings.

Our study extends a previous finding [12] of a significant interaction effect in children with physical–mental multimorbidity by examining a larger multimorbid sample across a broader range of mental health symptoms, and adjusting for more child, parent, family and social factors. Whilst we found a significant interaction effect, as per Sawyer et al. [12], our analyses revealed that this does not extend to borderline mental health symptoms, possibly due to the smaller span of scores within the borderline range compared with the clinical range of symptoms.

Our finding that the interaction effect of physical–mental multimorbidity was significant for boys but not girls is novel. This difference may arise due to our finding that the associations between physical and mental health problems and HRQoL were each larger and more significant for girls than boys; i.e. girls with physical health problems show significantly lower ratings of HRQoL compared to their healthy peers (regardless of their mental health status), however, for boys this was only true if they *also* had mental health problems. The differing relationships observed by sex may also relate to the type of mental health problems experienced by boys and girls, with internalising problems previously found to be related to poorer HRQoL in girls and externalising problems more-so for boys [20]. This warrants further exploration in the context of the interaction of physical–mental multimorbidity, to determine whether a particular combination of problems accounts for this difference.

Our sensitivity analysis found no interaction effect when using child self-reported HRQoL, which may be related to a number of factors. Firstly, parent ratings of children’s HRQoL tend to be lower than the child’s self-report when the child has chronic health conditions [8, 14, 40, 41], which may overestimate the deficit in HRQoL and lead to a stronger interaction effect of having multiple chronic conditions by parent-report. Secondly, in the current study, children and parents rated HRQoL using different measures. Whilst the PedsQL and CHU9D are moderately correlated (*r* = 0.63; when children self-report on both measures [42]), the use of different measures that comprise different domains of HRQoL, with different respondents makes it difficult to compare the findings. Similarly, the higher degree of variance in HRQoL scores that is explained using parent-report would likely be related to having the same respondent (parents) rate both the predictor (child’s health status) and outcome (HRQoL); i.e. part of this observed relationship reflects the parent’s underlying perception of the child’s health and wellbeing. Parent’s perception of their child’s health status can be further augmented by parents’ own mental health problems [43], which may also lead to the observed differences in child versus parent-report of HRQoL.

The overlap between measures of mental health and HRQoL is important to consider when assessing associations between the two [10, 37], though many studies have not addressed this [36]. Our finding that mental health problems were associated with larger differences in psychosocial domains of HRQoL is in line with a previous systematic review [11], however, this differs from others that have found stronger relationships in school functioning domains [14, 44]. Importantly, the relationship between mental health and HRQoL extended beyond the expected emotional health domain into all other domains examined. This broad relationship has previously been noted in studies using the PedsQL [9, 44], but is less consistently found using other measures [12–14]. Our findings add to a growing literature that mental health and HRQoL are related but distinct concepts [8].

In our adjusted model, only parental mental illness was associated with a clinically meaningful difference in the child’s HRQoL, holding all other factors constant. As the respondent in our study was the child’s mother in 98% of instances, our findings are similar to previous work by Bastiaansen et al. [20], which found that mother’s psychopathology was individually associated with poorer child HRQoL, though this was no longer significant in their final adjusted model. In contrast, recent evidence suggests parental mental illness may only be related to poorer HRQoL in the domain of autonomy & parent relation for adolescent girls, and may not be associated with any deficits in HRQoL for adolescent boys [45]. Whilst parental mental health may be an important, potentially modifiable, focus for intervention that may improve children’s HRQoL [20], further exploration is warranted particularly regarding the persistence of parental mental illness and links to children’s mental health problems as well as the implications for child self-report and parent-report of the child’s HRQoL over time.

### Strengths, limitations and recommendations for future research

This study presents the first evidence of the relative relationships between physical health, borderline and clinical levels of mental health symptoms and children’s HRQoL in a large, population-based sample. Using a population sample enabled us to detect a greater number of children within the borderline mental health range than might be possible with a clinical sample. The size of the sample enabled us to examine relationships within smaller subgroups of children, and relationships with a range of child, parent, family and social factors that have not been previously examined in this context.

Limitations include the use of parent-reported data on children’s physical and mental health problems rather than diagnosed conditions; in the absence of a gold-standard diagnostic interview, and the inconsistent availability of parent-reported mental health conditions across waves, we used the SDQ as a validated tool available at every time point. We recognise that use of the SDQ to measure mental health problems means not all children who screen positive for mental health problems would meet clinical criteria for a psychiatric diagnosis and, vice versa [46], some children with diagnosed conditions may be well-managed with treatment and may have low scores on the SDQ. This misalignment in identifying children with mental health problems can be seen within the current study regarding the differing results between SDQ and parent-report of children’s mental health diagnoses and speaks to the wider difficulty in accurately identifying this population. On the other hand, a strength of using the SDQ for all children meant we could identify children with borderline symptoms that may have gone undetected using formal clinical criteria. Using the same respondent for child mental health and HRQoL is a limitation which we have made efforts to address by including a range of parent factors as covariates in adjusted analyses, and conducted a sensitivity analysis using child self-reported HRQoL. An additional limitation arises from the pooled cross-sectional design of the study, such that we cannot comment on the direction of effect, and the analysis cannot control for unobserved factors (e.g. parent factors) that may impact reports of child HRQoL and child health. Whilst it seems likely that physical health problems would cause poorer HRQoL, it is not necessarily the case that the relationship between mental health problems and HRQoL is unidirectional. A longitudinal analysis is planned to examine this interplay of symptoms, as well as the potentially changing influence of child, parent, family and social factors, on children’s HRQoL throughout childhood and adolescence.

### Clinical implications

Given the relationship between greater mental health symptoms and poorer HRQoL in children of all ages, clinicians should seek to identify mental health symptoms in children as young as 4–7 years old, even if these problems would not meet formal clinical criteria for a mental health disorder. Particular attention should be paid to the mental health and HRQoL of children, especially boys, with physical–mental multimorbidity. Maternal mental illness may represent a potentially modifiable area for intervention alongside treatment of the child’s symptoms. Siloing of child and adult mental health services may mean that this does not happen in practice, though this has been trialled in the United Kingdom [47].

### Conclusion

Children with physical–mental multimorbidity are at risk of disproportionately poorer HRQoL than the additive effect of both problems. Our results suggest that these children are a group with particularly high needs who would benefit from health policy and clinical decision making to address these needs. Similarly, if we are to improve children’s HRQoL, monitoring and addressing borderline mental health symptoms in children as young as 4–7 years old is important but not happening in Australia [48].

## Supplementary Information

Below is the link to the electronic supplementary material.

Supplementary file1 (DOCX 94 kb)

## Data Availability

Study data are freely available and can be requested from the data custodians, the Australian Data Archive (ADA) in collaboration with the National Centre for Longitudinal Data (NCLD, Australian Government Department of Social Services). Access available via Dataverse.

## Code availability

Data analysis code is available upon request (Stata/SE v.16.0).
