# Supplementary material for: The relationship between physical and mental health multimorbidity and children’s health-related quality of life
Source: Qual Life Res. 2022 Jan 29;31(7):2119–31. doi: 10.1007/s11136-022-03095-1 (PMC9188523; doi:10.1007/s11136-022-03095-1)
Supplement: Supplementary file 1 — Supplementary file1 (DOCX 94 kb) [file 11136_2022_3095_MOESM1_ESM.docx]

**SUPPLEMENTARY MATERIAL**

**Suppl. Table 1. Full details of covariates included in adjusted analyses.**

| **Construct** | **Specification** | **Justification for inclusion** |
| --- | --- | --- |
| *Child age* | Recorded at each wave and included in analyses as a categorical variable where 1 = ‘4-7 years’; 2 = ‘8-12 years’ and 3 = ‘13-17 years’. | Age was included as a categorical variable, as (1) this aligns with the use of the various developmentally appropriate forms of the PedsQL in each of these age bands; and (2) as per all other covariates, to examine the relationship between each age group and HRQoL. These categories provide informative comparisons between preschool, primary school and high-school aged children rather than changes in HRQoL related to annual increases in age. |
| *Child sex* | Recorded in LSAC only at Wave 1, and this value was continued to other waves. Coded as 0 = ‘male’ and 1 = ‘female’. | Included as a child-specific factor. |
| *Child ethnicity* | Measured as the main language spoken by the child at home. Dichotomised and coded as 0 = ‘English’; 1 = ‘Language other than English (LOTE)’. | Ethnicity has been linked to differences in children’s HRQoL [1] and, within the Bronfenbrenner model [2], forms an important part of the sociocultural influences on children’s lives. |
| *Parent education* | Measured as primary carer completion of university or TAFE. Binary variable coded as 0 = ‘no’; 1 = ‘yes’. | Included as a family-level indicator of socioeconomic status. |
| *Single parent households* | Parent reported at each Wave. Included as a dichotomised variable, coded as 0 = ‘households with two parents (either biological or non-biological)’; and 1 = ‘single parent households (either biological or non-biological)’. | Previously included when examining factors related to children’s HRQoL over-and-above mental health symptoms [3]. |
| *Number of siblings in household* | Parent reported at each Wave. Dichotomised and coded as 0 = ‘0 or 1 sibling(s)’; 1 = ‘2 or more siblings’. | Included as a family level factor based on the Bronfenbrenner model [2]. |
| *Parent mental illness* | Measured for the primary carer using the Kessler 6 screening scale [4]; a six item scale that asks “In the past four weeks, about how often did you feel… nervous”, “hopeless”, “restless or fidgety”, “that everything was an effort”, “so sad that nothing could cheer you up”, and “worthless”, scored from 1 ‘all the time’ to 5 ‘none of the time’. Scores were reverse coded such that higher scores reflect greater problems. Total scores were dichotomised in line with the recommended clinical cut-points of 0 = ‘Score of 6-18, No probable serious mental illness’; 1 = ‘Score of ≥19, Probable serious mental illness’. | Included in previous research [3] as a potentially modifiable area for intervention that can more holistically improve children’s HRQoL alongside treatment of the child’s symptoms. |
| *Maladaptive parenting* | Characterised by high hostility and low warmth, maladaptive parenting was included as a dichotomous variable. As per existing literature using LSAC data, *parental warmth* was measured as the mean of six items, e.g. “How often do you… express affection by hugging, kissing and holding this child”, “tell this child how happy he/she makes you”. Secondly, *parental hostility* was measured as the mean of six items, e.g. “How often do you… get angry when you punish this child”, “tell this child that he/she is bad or not as good as others”. Both are rated on a 5-point scale from 1 ‘never/almost never’ to 5 ‘all the time’, such that higher scores represent higher levels of parenting warmth and hostility. Responses were divided into quintiles, and those who scored in the highest quintile for *hostility* and the lowest quintile for *warmth* were coded as 1 = ‘maladaptive parenting’; all other response combinations were coded as 0 = ‘not maladaptive parenting’. | Maladaptive parenting, coded in this way, has been used in previous LSAC research and linked to greater emotional symptoms in children [5]. A similar family functioning variable has previously been linked to poorer child HRQoL [3]. |
| *Annual household income* | Parents reported household income at each wave as the combined income of all adults in the household. Where possible, missing income values were imputed by the LSAC team; the imputation methods employed are described in detail elsewhere [6]. Values were categorised based on the 2021 Australian Taxation Office income brackets, and coded as 1 = ‘$0-$18,200’; 2 = ‘$18,201-$45,000’; 3 = ‘$45,001-$120,000’; 4 = ‘$120,001-$180,000’; 5 = ‘$180,001 and over’. | Included as a family-level indicator of socioeconomic status. |
| *Socioeconomic status* | Socioeconomic status was estimated using the Socio-Economic Index for Areas (SEIFA) score, specifically, the Index of Relative Socio-Economic Advantage and Disadvantage (IRSAD) [7]. This score is assigned for every Australian postcode based on census data reflecting the economic and social conditions of people and households within an area. It is designed to have a national mean of 1,000 and SD of 100, where a lower score represents greater disadvantage and a higher score represents greater advantage. SEIFA IRSAD scores were classified into quintiles for use in analyses. | Included as a neighbourhood-level indicator of socioeconomic status. The IRSAD index is recommended when the topic being analysed is likely to be affected by both advantage and disadvantage, not just by presence and absence of disadvantage. |
| *Rurality* | Rurality of the child’s home postcode was dichotomised as 1 = ‘Major Cities of Australia’ and 0 = ‘Other’, which included all other categories (i.e. regional, rural etc.) in line with the Australian Statistical Geography Standard levels of remoteness [8]. | Disparities in health and development are known to exist across metropolitan and rural/regional/remote areas of Australia [9]. It is hypothesised that this extends to HRQoL. |

**Suppl. Table 2. Details of sensitivity analyses performed.**

| **Construct** | **Alternate Specifications** |
| --- | --- |
| *Physical health* | (1) Any chronic physical health condition, regardless of special healthcare needs. Binary variable coded as 0 = ‘no’; 1 = ‘yes’. |
|  | (2) Total number of chronic health conditions. Dichotomised and coded as 0 = ‘0-2 conditions’; 1 = ‘3 or more conditions’. |
| *Mental health* | In low risk community samples, two composite subscales are commonly used to measure ‘internalising problems’ (peer and emotional subscales); and ‘externalising problems’ (conduct and hyperactivity subscales) [10]. Based on Australian norms [11], for the internalising and externalising subscales, scores of ≥9 and ≥10 (out of 20), respectively, indicate clinically elevated symptoms. Alternate definitions of mental health status included as sensitivity analyses were:  (1) Internalising problems only. Dichotomised using the clinical cut-off for the SDQ internalising subscale. Binary variable coded as 0 = ‘<9 subscale score’; 1 = ‘9+ subscale score’. |
|  | (2) Externalising problems only. Dichotomised using the clinical cut-off for the SDQ externalising subscale. Binary variable coded as 0 = ‘<10 subscale score’; 1 = ‘10+ subscale score’. |
|  | (3) Parent report of the child’s anxiety and/or depression, not required to be diagnosed by a clinician. Binary variable coded as 0 = ‘no’; 1 = ‘yes’. Variable is available in the LSAC dataset in various forms (i.e. combined report for anxiety/depression; or individual report of anxiety; individual report of depression), and not available in every wave.  (4) Parent report of the child’s Attention-Deficit/Hyperactivity Disorder (ADHD), not required to be diagnosed by a clinician. Binary variable coded as 0 = ‘no; 1 = ‘yes’. Variable is available in the LSAC dataset as one question, available in all waves. |
| *Health-related quality of life (HRQoL)* | (1) PedsQL total score excluding emotional subscale items due to item overlap with mental health measurement. Replaces PedsQL total score as outcome measure. Retains 0-100 scaling of original measure. |
|  | (2) Child self-reported HRQoL using the Child Health Utility instrument (CHU9D); a 9-item measure that assesses the child’s functioning ‘today’. The CHU9D has shown to be feasible and valid in a sample of Australian adolescents aged 11-17 years [12], and was included in the LSAC data collection for older children, for Waves 6-8. CHU9D utility scores range from 0-1 where higher scores represent better HRQoL. |

**Suppl. Table 3. Data tables for Figure 1.**

|  |  | **PH*MH Interaction** | | | **Physical health (with SHCN)** | | |
| --- | --- | --- | --- | --- | --- | --- | --- |
|  |  | *Coef.* | *95%CI* | *p* | *Coef.* | *95%CI* | *p* |
| **Base Case** | Base case | **-3.6** | **-4.8 to -2.4** | **<0.0001** | **-4.4** | **-4.9 to -4.0** | **<0.0001** |
| **Child Age** | 4-7 years | -3.9 | -6.2 to -1.6 | 0.001 | **-3.8** | **-4.6 to -3.0** | **<0.0001** |
|  | 8-12 years | -2.5 | -4.3 to -0.7 | 0.006 | **-3.9** | **-4.5 to -3.2** | **<0.0001** |
|  | 13-17 years | -2.7 | -5.1 to -0.2 | 0.031 | **-5.6** | **-6.5 to -4.7** | **<0.0001** |
| **Child Sex** | Male | **-4.8** | **-6.4 to -3.1** | **<0.0001** | **-4.0** | **-4.6 to -3.4** | **<0.0001** |
|  | Female | -2.3 | -4.2 to -0.3 | 0.025 | **-4.8** | **-5.5 to -4.1** | **<0.0001** |
| **PH Defs** | Any chronic PH condition (y/n) | **-5.1** | **-6.7 to -3.5** | **<0.0001** | **-2.5** | **-2.8 to -2.2** | **<0.0001** |
|  | Number of conditions (0-2; 3+) | **-4.4** | **-5.6 to -3.2** | **<0.0001** | **-4.5** | **-4.9 to -4.0** | **<0.0001** |
| **MH Defs** | SDQ internalising subscale cut-off, (<9 vs 9+) | **-2.9** | **-4.2 to -1.7** | **<0.0001** | **-5.5** | **-6.0 to -5.1** | **<0.0001** |
|  | SDQ externalising cut-off, (<10 vs 10+) | **-2.9** | **-4.1 to -1.7** | **<0.0001** | **-6.7** | **-7.1 to -6.2** | **<0.0001** |
|  | Parent reported anxiety /depression (y/n) | -1.8 | -3.5 to -0.1 | 0.036 | **-6.3** | **-6.9 to -5.8** | **<0.0001** |
|  | Parent reported ADHD (y/n) | 0.6 | -1.8 to 2.9 | 0.622 | **-7.5** | **-8.0 to -7.0** | **<0.0001** |
| **QoL Defs** | PedsQL minus emotional scale | **-2.9** | **-4.2 to -1.5** | **<0.0001** | **-5.3** | **-5.8 to -4.8** | **<0.0001** |
|  |  |  |  |  |  |  |  |
|  |  | **Mental health (borderline)** | | | **Mental health (clinical)** | | |
|  |  | *Coef.* | *95%CI* | *p* | *Coef.* | *95%CI* | *p* |
| **Base Case** | Base case | **-10.5** | **-11.0 to -9.9** | **<0.0001** | **-16.8** | **-17.6 to -16.1** | **<0.0001** |
| **Child Age** | 4-7 years | **-7.6** | **-8.4 to -6.8** | **<0.0001** | **-12.1** | **-13.4 to -10.9** | **<0.0001** |
|  | 8-12 years | **-11.5** | **-12.3 to -10.7** | **<0.0001** | **-18.7** | **-19.9 to -17.5** | **<0.0001** |
|  | 13-17 years | **-12.5** | **-13.7 to -11.3** | **<0.0001** | **-19.6** | **-21.2 to -17.9** | **<0.0001** |
| **Child Sex** | Male | **-9.4** | **-10.1 to -8.8** | **<0.0001** | **-16.3** | **-17.4 to -15.3** | **<0.0001** |
|  | Female | **-11.6** | **-12.4 to -10.8** | **<0.0001** | **-17.3** | **-18.4 to -16.1** | **<0.0001** |
| **PH Defs** | Any chronic PH condition (y/n) | **-9.4** | **-10.4 to -8.4** | **<0.0001** | **-14.9** | **-16.4 to -13.4** | **<0.0001** |
|  | Number of conditions (0-2; 3+) | **-10.2** | **-10.7 to -9.6** | **<0.0001** | **-17.0** | **-17.7 to -16.3** | **<0.0001** |
| **MH Defs** | SDQ internalising subscale cut-off, (<9 vs 9+) | *N/A* | *N/A* | *N/A* | **-16.6** | **-17.3 to -15.8** | **<0.0001** |
|  | SDQ externalising cut-off, (<10 vs 10+) | **-8.5** | **-9.2 to -7.9** | **<0.0001** | *N/A* | *N/A* | *N/A* |
|  | Parent reported anxiety /depression (y/n) | **-11.5** | **-12.6 to -10.4** | **<0.0001** | *N/A* | *N/A* | *N/A* |
|  | Parent reported ADHD (y/n) | **-7.4** | **-9.4 to -5.4** | **<0.0001** | *N/A* | *N/A* | *N/A* |
| **QoL Defs** | PedsQL minus emotional scale | **-9.1** | **-9.7 to -8.6** | **<0.0001** | **-15.1** | **-15.9 to -14.3** | **<0.0001** |

NB: Bolding denotes statistically significant effects at *p* <.0001; Shading denotes statistically significant and clinically meaningful differences.

**Suppl. Table 4. Adjusted regression results for PedsQL total score across age bands and child sex**

|  | **Age bands** | | | | | | **Child sex** | | | |
| --- | --- | --- | --- | --- | --- | --- | --- | --- | --- | --- |
|  | 4-7 years | | 8-12 years | | 13-17 years | | Male | | Female | |
|  | *Coef.* | *p* | *Coef.* | *p* | *Coef.* | *p* | *Coef.* | *p* | *Coef.* | *p* |
| **Physical health** |  |  |  |  |  |  |  |  |  |  |
| Physical health, *ref ‘no PH’* | **-3.8** | **<.0001** | **-3.9** | **<.0001** | **-5.6** | **<.0001** | **-4.0** | **<.0001** | **-4.8** | **<.0001** |
| **Mental health *(ref ‘<13’)*** |  |  |  |  |  |  |  |  |  |  |
| Borderline *(SDQ total = 13-16)* | **-7.6** | **<.0001** | **-11.5** | **<.0001** | **-12.5** | **<.0001** | **-9.4** | **<.0001** | **-11.6** | **<.0001** |
| Clinical *(SDQ total = 17+)* | **-12.1** | **<.0001** | **-18.7** | **<.0001** | **-19.6** | **<.0001** | **-16.3** | **<.0001** | **-17.3** | **<.0001** |
| **PH * MH *(ref ‘none’)*** |  |  |  |  |  |  |  |  |  |  |
| PH * Borderline MH | 0.6 | 0.560 | -1.0 | 0.268 | 1.0 | 0.396 | -0.7 | 0.366 | -0.1 | 0.929 |
| PH * Clinical MH | -3.9 | 0.001 | -2.5 | 0.006 | -2.7 | 0.031 | **-4.8** | **<.0001** | -2.3 | 0.025 |
| Regression constant | 84.1 | <.0001 | 80.2 | <.0001 | 79.7 | <.0001 | 80.5 | <.0001 | 82.5 | <.0001 |
| R-squared | 0.18 | | 0.29 | | 0.31 | | 0.28 | | 0.25 | |
| Number of obs. | 14,315 | | 18,405 | | 10,917 | | 22,324 | |  | |

MH = mental health; PH = physical health; SDQ = Strengths and Difficulties Questionnaire

NB: All analyses in this table are adjusted for the same covariates as described in the adjusted analyses in the main text. Results for covariate coefficients not displayed for simplicity of reading.

Bolding denotes statistically significant effects at *p* <.0001; Shading denotes statistically significant and clinically meaningful differences.

**Suppl. Table 5. Adjusted regression results for PedsQL domain****s individually**

|  | **PedsQL Domains** | | | | | | | |
| --- | --- | --- | --- | --- | --- | --- | --- | --- |
|  | Physical Health^a^ | | Emotional Functioning^b^ | | Social Functioning^c^ | | School Functioning^d^ | |
|  | *Coef.* | *p* | *Coef.* | *p* | *Coef.* | *p* | *Coef.* | *p* |
| **Physical health** |  |  |  |  |  |  |  |  |
| Physical health, *ref ‘no PH’* | **-4.4** | **<.0001** | **-3.9** | **<.0001** | **-3.6** | **<.0001** | **-7.9** | **<.0001** |
| **Mental health *(ref ‘<13’)*** |  |  |  |  |  |  |  |  |
| Borderline *(SDQ total = 13-16)* | **-7.7** | **<.0001** | **-13.7** | **<.0001** | **-13.3** | **<.0001** | **-6.4** | **<.0001** |
| Clinical *(SDQ total = 17+)* | **-12.8** | **<.0001** | **-20.4** | **<.0001** | **-22.8** | **<.0001** | **-9.7** | **<0.001** |
| **PH * MH *(ref ‘none’)*** |  |  |  |  |  |  |  |  |
| PH * Borderline MH | 0.8 | 0.359 | -0.4 | 0.034 | -1.4 | 0.093 | -1.6 | 0.106 |
| PH * Clinical MH | -0.5 | 0.601 | **-6.5** | **<.0001** | **-6.4** | **<.0001** | -1.9 | 0.060 |
| Regression constant | 82.6 | <.0001 | 78.4 | <.0001 | 84.1 | <.0001 | 90.9 | <.0001 |
| R-squared | 0.12 | | 0.26 | | 0.24 | | 0.13 | |
| Number of obs. | 43,618 | | 43,631 | | 43,550 | | 43,614 | |

MH = mental health; PH = physical health; SDQ = Strengths and Difficulties Questionnaire

NB: All analyses in this table are adjusted for the same covariates as described in the adjusted analyses in the main text. Results for covariate coefficients not displayed for simplicity of reading.

Bolding denotes statistically significant effects at *p* <.0001; Shading denotes statistically significant and clinically meaningful differences.

^a^ Minimal clinically important difference for physical health subscale is 6.92 [13]

^b^ Minimal clinically important difference for emotional functioning subscale is 7.79 [13]

^c^ Minimal clinically important difference for social functioning subscale is 8.98 [13]

^d^ Minimal clinically important difference for school functioning subscale is 9.67 [13]

**Suppl. Table 6. Sensitivity analysis results for physical, mental and interaction effects using HRQoL outcome defined as child self-reported CHU9D utility score.**

|  | **HRQoL Definition** | |
| --- | --- | --- |
|  | CHU9D | |
|  | *Coef.* | *p* |
| **Physical health** |  |  |
| Physical health, *ref ‘no PH’* | **-0.03** | **<.0001** |
| **Mental health *(ref ‘<13’)*** |  |  |
| Borderline *(SDQ total = 13-16)* | **-0.05** | **<.0001** |
| Clinical *(SDQ total = 17+)* | **-0.08** | **<.0001** |
| **PH * MH *(ref ‘none’)*** |  |  |
| PH * Borderline MH | 0.001 | 0.964 |
| PH * Clinical MH | -0.01 | 0.501 |
| Regression constant | 0.87 | <.0001 |
| R-squared | 0.07 | |
| Number of obs. | 14,041 | |

MH = mental health; PH = physical health; SDQ = Strengths and Difficulties Questionnaire

NB: All analyses in this table are adjusted for the same covariates as described in the adjusted analyses in the main text. Results for covariate coefficients not displayed for simplicity of reading.

Bolding denotes statistically significant effects at *p* <.0001; No shading as per previous tables – clinically meaningful difference discussed for PedsQL does not apply to CHU9D as outcome.

**Supp. Figure 1. Participant Flowchart.**

**
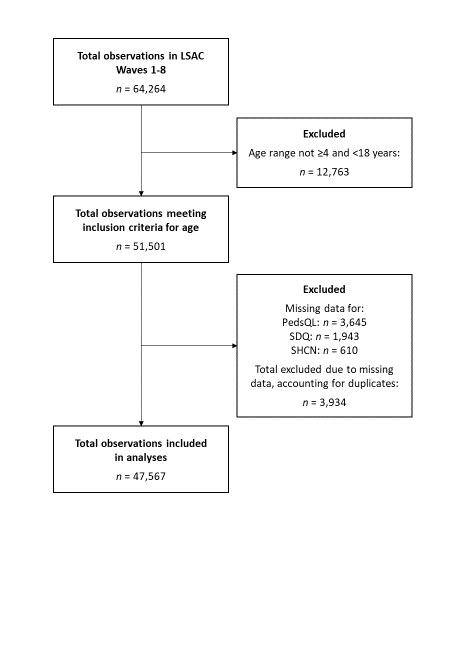
**

**LSAC ‘Longitudinal Study of Australian Children’; PedsQL ‘Pediatric Quality of Life Inventory’; SDQ ‘ Strengths and Difficulties Questionnaire’; SHCN ‘Special Healthcare Needs’.**

**Supp. Figure 2. Interaction effect of physical health problems and mental health symptoms on health-related quality of life for boys versus girls**

These figures show the main effects and interaction effects between physical health problems and mental health symptoms and children’s health-related quality of life (HRQoL), for boys and girls separately.

Main effects for boys and girls are: increasing mental health symptoms (along the x-axis) are associated with poorer HRQoL (on the y-axis); and the presence of physical health problems plus special healthcare needs (SHCN; dotted lines) is associated with poorer HRQoL than children without physical health problems (solid lines).

The interaction effect between physical health and mental health is shown in the difference in slope of the two lines. The interaction was not significant for girls; shown visually with more parallel lines, meaning the difference in QoL between girls who don’t have physical health problems and those who do have problems is the same, no matter what level of mental health symptoms the child has.

The interaction was significant for boys; shown visually with difference in slope between the groups. This means – for boys – when mental health symptoms are very low, there is little difference in HRQoL between boys who do and don’t have physical health problems. However, as boys’ mental health symptoms become greater, the difference between these groups becomes greater and greater. This interaction says that, for boys, the decrement in HRQoL associated with physical-mental multimorbidity depends on the level of mental health symptoms.

*[GIRLS] [BOYS]*

**References for Supplementary Material**

1. Wallander, J. L., Fradkin, C., Elliott, M. N., Cuccaro, P. M., Tortolero Emery, S., & Schuster, M. A. (2019). Racial/ethnic disparities in health-related quality of life and health status across pre-, early-, and mid-adolescence: a prospective cohort study. *Quality of Life Research*, *28*(7), 1761–71. https://doi.org/10.1007/s11136-019-02157-1

2. Bronfenbrenner, U. (1979). *The ecology of human development: Experiments by nature and design.* Cambridge, MA: Harvard University Press.

3. Bastiaansen, D., Koot, H. M., & Ferdinand, R. F. (2005). Determinants of quality of life in children with psychiatric disorders. *Quality of Life Research*, *14*(6), 1599–1612. https://doi.org/10.1007/s11136-004-7711-2

4. Kessler, R. C., Barker, P. R., Colpe, L. J., Epstein, J. F., Gfroerer, J. C., Hiripi, E., … Zaslavsky, A. M. (2003). Screening for serious mental illness in the general population. *Archives of General Psychiatry*, *60*(2), 184–9. https://doi.org/10.1001/archpsyc.60.2.184

5. Farrant, B. (2014). Maladaptive parenting and child emotional symptoms in the early school years: Findings from the Longitudinal Study of Australian Children. *Australasian Journal of Early Childhood*, *39*(2), 118–125. https://doi.org/10.1177/183693911403900215

6. Mullan, K., Daraganova, G., & Baker, K. (2015). *Growing Up in Australia : The Longitudinal Study of Australian Children (LSAC). LSAC Technical Paper #14. Imputing income in the Longitudinal Study of Australian Children.* Canberra, Australia.

7. Australian Bureau of Statistics. (2018). 2033.0.55.001 - Census of Population and Housing: Socio-Economic Indexes for Areas (SEIFA), Australia, 2016. *Canberra: Australian Bureau of Statistics*. Retrieved March 23, 2021, from https://www.abs.gov.au/ausstats/abs@.nsf/Lookup/by Subject/2033.0.55.001~2016~Main Features~IRSAD~20

8. Australian Bureau of Statistics. (2018). 1270.0.55.005 - Australian Statistical Geography Standard (ASGS): Volume 5 - Remoteness Structure, July 2016. Table 3: Correspondence 2017 Postcode to 2016 Remoteness Area. *Canberra: Australian Bureau of Statistics*. Retrieved March 23, 2021, from https://www.abs.gov.au/AUSSTATS/abs@.nsf/DetailsPage/1270.0.55.005July 2016?OpenDocument

9. Arefadib, N., & Moore, T. (2017). *Reporting the Health and Development of Children in Rural and Remote Australia. Report for the Royal Far West.* Parkville.

10. Goodman, A., Lamping, D. L., & Ploubidis, G. B. (2010). When to use broader internalising and externalising subscales instead of the hypothesised five subscales on the strengths and difficulties questionnaire (SDQ): Data from british parents, teachers and children. *Journal of Abnormal Child Psychology*, *38*(8), 1179–91. https://doi.org/10.1007/s10802-010-9434-x

11. Mellor, D. (2005). Normative data for the Strengths and Difficulties Questionnaire in Australia. *Australian Psychologist*, *40*(3), 215–22. https://doi.org/10.1080/00050060500243475

12. Stevens, K., & Ratcliffe, J. (2012). Measuring and Valuing Health Benefits for Economic Evaluation in Adolescence: An Assessment of the Practicality and Validity of the Child Health Utility 9D in the Australian Adolescent Population. *Value in Health*, *15*(1), 1092–9. https://doi.org/10.1016/j.jval.2012.07.011

13. Varni, J. W., Burwinkle, T. M., Seid, M., & Skarr, D. (2003). The PedsQL^TM^* 4.0 as a pediatric population health measure: Feasibility, reliability, and validity. *Ambulatory Pediatrics*, *3*(6), 329–341. https://doi.org/10.1367/1539-4409(2003)003<0329:TPAAPP>2.0.CO;2
